# Emission characteristics and health effects of PM_2.5_ from vehicles in typical areas

**DOI:** 10.3389/fpubh.2024.1326659

**Published:** 2024-06-19

**Authors:** Xiaowei Song, Yongpei Hao

**Affiliations:** College of Resources and Environment, Shanxi University of Finance and Economics, Taiyuan, China

**Keywords:** PM_2.5_, vehicular pollution, health risk, economic loss, typical urban agglomerations

## Abstract

**Introduction:**

Vehicle emissions have become an important source of urban air pollution, and the assessment of air pollution emission characteristics and health effects caused by specific pollution sources can provide scientific basis for air quality management.

**Methods:**

In this paper, vehicle PM_2.5_ pollution in typical urban agglomerations of China (the Beijing-Tianjin-Hebei urban agglomeration (BTHUA), the triangle of the Central China urban agglomeration (TCCUA) and the Chengdu-Chongqing urban agglomeration (CCUA)) were used as research samples to evaluate the emission characteristics, health effects and economic losses of vehicle PM_2.5_ pollution based on the emission inventory, air quality model and exposure-response function from 2010 to 2020.

**Results:**

The results indicated that PM_2.5_ emissions from vehicles in the three urban agglomerations during 2010-2020 first showed an upward yearly trend and then showed a slow decrease in recent years. Heavy-duty trucks and buses are the main contribution vehicles of PM_2.5_, and the contribution rates of light-duty vehicles to PM_2.5_ is increasing year by year. The contribution rate of PM_2.5_ in Beijing decreased significantly. In addition to capital cities and municipalities directly under the central Government, the emission of pollutants in other cities cannot be ignored. The evaluation results of the impact of PM_2.5_ pollution from vehicles on population health show that: the number of each health endpoint caused by PM_2.5_ pollution from vehicles in the BTHUA and CCUA showed an overall upward trend, while the TCCUA showed a downward trend in recent years. Among them, PM_2.5_ pollution from vehicles in the three major urban agglomerations cause about 78,200 (95% CI: 20,500-138,800) premature deaths, 122,800 (95% CI: 25,600-220,500) inpatients, and 628,400 (95% CI: 307,400-930,400) outpatients and 1,332,400 (95% CI: 482,700-2,075,600) illness in 2020. The total health economic losses caused by PM_2.5_ pollution from vehicles in the three major urban agglomerations in 2010, 2015 and 2020 were 68.25 billion yuan (95% CI: 21.65-109.16), 206.33 billion yuan (95% CI: 66.20-326.20) and 300.73 billion yuan (95% CI: 96.79-473.16), accounting for 0.67% (95% CI: 0.21-1.07%), 1.19% (95% CI: 0.38%-1.88%) and 1.21% (95% CI: 0.39%-1.90%) of the total GDP of these cities.

**Discussion:**

Due to the differences in vehicle population, PM_2.5_ concentration, population number and economic value of health terminal units, there are differences in health effects and economic losses among different cities in different regions. Among them, the problems of health risks and economic losses were relatively prominent in Beijing, Chengdu, Chongqing, Tianjin and Wuhan.

## 1 Introduction

With the rapid development of global economy, the numbers of vehicles have increased rapidly in China. Vehicular pollution has become an important sources of air pollution, seriously affecting climate change and human health (1–4). In particular, PM_2.5_, as a major pollutant in the current atmospheric environment, has had a serious negative impact on people’s lives and physical and mental health (5–7), increasing the risk of diseases and restricting the sustainable development of society. Although the time people are exposed to the traffic environment is very short every day, the proportion of their exposure dose in the total exposure amount is high (8). Especially during rush hour, when the motor vehicle is running at low speed or idling, pollutant emissions are large, and the harm to the human body is greater (9). In 2015, 385 (95% CI: 274–493) 1,000 deaths worldwide were linked to transport air pollution emissions, accounting for 11.4% of the global air pollution disease burden. In 2018, the number of all-cause premature deaths caused by PM_2.5_ emissions in China’s transportation sector was 149,000, including 159,100 inpatients for cardiovascular diseases, 103,700 inpatients for respiratory diseases, 6,416,900 outpatients for cardiovascular diseases, and 6,309,900 outpatients for respiratory diseases. The health and economic losses caused by pollutant emissions from the transportation sector reached 347.942 billion yuan, accounting for approximately 0.39% of China’s GDP over the years (10).

To promote vehicle pollution reduction, many studies have used different methods to study the estimation and control strategies of vehicular emissions in some areas (11–13). For example, some Chinese scholars have studied the emission inventories and characteristics of vehicular pollutants in economically developed urban agglomerations in eastern China (14–17). In addition, the effect of vehicle emissions reduction programs has been evaluated based on emission inventories (15–19).

Epidemiological studies have shown that PM_2.5_ is the air pollutant most closely related to health. The developed countries started research on the effects of air pollution earlier, and the research methods and research systems are relatively perfect. For example, the U.S. Environmental Protection Agency (USEPA) developed the Environmental Benefits Mapping and Analysis Program (BenMap) model for health benefit assessment, which is now widely used worldwide (20–22). Buekers et al. (23) used the Extreme E model to assess the health benefits from the introduction of electric vehicles in various European countries (23). In China, there has been increasing research on the health effects of air pollution in recent years (24–27). Zeng et al. (28) estimated the disease burden caused by PM_2.5_ pollution in 2017 in China and found that the average annual labor loss caused by PM_2.5_ pollution in China was 25,903,400 d, the additional medical expenditure of residents was 8,639 billion yuan, and the economic loss accounted for approximately 1.48% of the GDP of that year. However, there are relatively few researches on the health effects of air pollution from specific pollution sources, and assessing the health losses caused by air pollution from specific pollution sources is the key for government departments to carry out cost–benefit analysis on various policy measures and optimize pollution prevention and control strategies.

In reality, different regions differ greatly in economic development, energy composition and transportation structure configuration. For example, the Beijing–Tianjin–Hebei urban agglomeration is the most economically developed typical region in China, and its vehicle ownership is larger than that of other regions, resulting in significant differences in vehicle pollutant emissions. The geographical location and topographic characteristics of different regions also have an impact on the emission characteristics of vehicles. For example, there is a great deal of mountainous terrain in the Chengdu–Chongqing urban agglomeration, which increase the number of uphill climbs for vehicles, resulting in increased pollutant emissions. In addition, the relevant measures taken by various regions to promote the emission reduction of vehicle pollutants are different. From the current situation of promoting new-energy vehicles in various regions of China, regional differences are very obvious, which leads to different abilities and measures to reduce air pollutants in different regions. The cost of reducing environmental pollution as well as the health effects and economic losses caused by pollution in economically developed regions and less developed regions are also different. However, researchers focused more on vehicular emissions in China’s developed regions and cities, such as the Yangtze River Delta, the Beijing–Tianjin–Hebei region, and Guangdong (14, 16, 17). Moreover, studies on the health effects and economic losses caused by regional vehicle pollution are lacking, as well as comparisons between urban agglomerations of different economic development scales and geographical locations.

Therefore, in order to reasonably assess the status and change trend of regional vehicle pollution emissions and clarify the extent of their impact on residents’ health in different regions and economic losses, this paper establishes the emission inventory of vehicle pollutants PM_2.5_ in the Beijing–Tianjin–Hebei urban agglomeration (BTHUA), the triangle of the Central China urban agglomeration (TCCUA) and the Chengdu–Chongqing urban agglomeration (CCUA) from 2010 to 2020. This study shows the characteristics such as time variation of total vehicle pollutant emission and contribution rates by different vehicle types and cities, analyzes the health effect and economic loss caused by PM_2.5_ pollution, and compares the assessment results in different cities and regions. The results can provide some reference for effectively controlling the environmental health impacts caused by vehicle pollution and help environmental protection authorities implement emission reduction measures according to local conditions, effectively reduce the burden of health diseases caused by vehicle pollution emissions, and maximize the health benefits of emission reduction policies.

## 2 Methodology

### 2.1 Emission estimates

**Figure 1:**
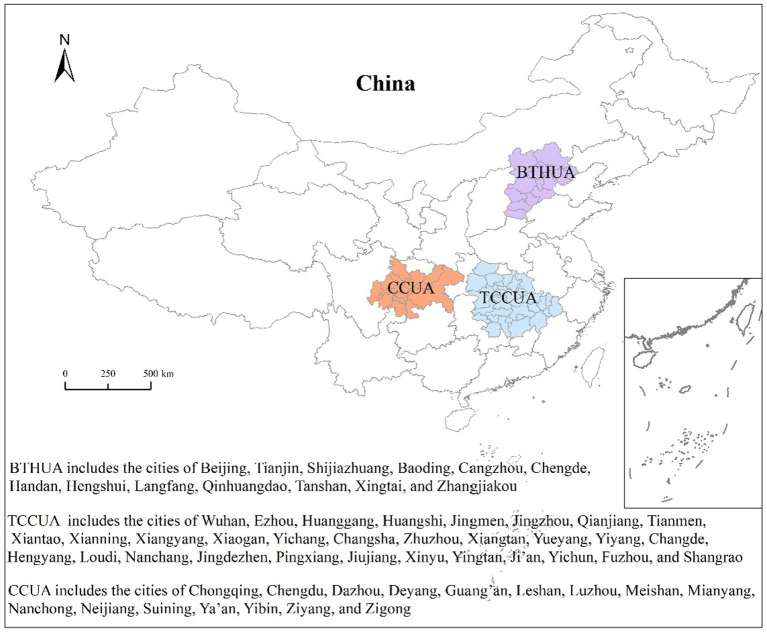
The geographical locations of the BTHUA, TCCUA, and CCUA.

Vehicular emissions can be estimated by the following Formula (1) (29), as follows:


(1) $$T_{n}=\underset{q}{\sum }\underset{p}{\sum }\left(V_{n,q,p}\times VKT_{n,q}\times EF_{q,p}\right)$$

where *T_n_* is the annual emissions of vehicle pollutants in area *n* (g), *V_n,q,p_* refers to the vehicle population in category *q* with emission standard *p* in area *n*, *VKT_n,q_* refers to the average annual VKTs for vehicles in category *q* in area *n* (km), and *EF_p,q_* is the emission factor for vehicle pollutants in category *q* with emission standard *p* (g/km). In the formula, *n* refers to 60 cities in the three urban agglomerations (the geographical locations and cities of the three urban agglomerations are shown in Figure 1), *q* represents the category of vehicles [including passenger cars (PCs), buses (BUSs), light-duty vehicles (LDVs), heavy-duty trucks (HDTs), and motorcycles (MCs)], and *p* is the emission standards implemented for different vehicle types during 2010–2020 (Supplementary Table A1).

#### 2.1.1 Vehicle population

The vehicle populations in the 60 cities in the three urban agglomerations were obtained from the statistical yearbooks of revenant provinces and cities and the China automotive industry yearbook from 2011 to 2021. This study assumes that newly produced vehicles must comply with new emission standards once they are enacted (30–32). The vehicle populations with different emission standards for different types are established by the Formula (2) referred to Lang et al. (29) and Sun et al. (31), as follows:


(2) $$Q_{a,m,n}=\left\{ \begin{array}{l} \underset{b}{\sum }T_{a,m,n}\cdot r_{y-k}\;n\neq S0\\ Q_{a,m,total}-\overset{S6}{\underset{S1}{\sum }}\underset{n}{\sum }T_{a,m,n}\cdot u_{y-k}\;n=S0 \end{array}\right.$$

where *T_a,m,n_* refers to the number of new registrations of vehicle *m* implementing emission standard *n* in city *a* (S0, S1 and S6 refers to the emission standards of State 0, State I and State VI, respectively), *Q_a,m,total_* refers to the total vehicle population *m* in city *a*, *k* refers to the year of vehicle registration, *y* is the study year, *b* is the duration of implementation of emission standard *n*, and *u* is the vehicle survival rate which refer to the relevant literatures (1, 29, 31, 33) and are summarized in Supplementary Table A2.

#### 2.1.2 Vehicle kilometers traveled (VKTs)

Data on the average annual VKTs of different vehicle types in the three urban agglomerations from 2010 to 2020 were based on the literature (3, 14, 25, 34–36) and referred to the data in cities with different development levels in China (2, 29, 33, 37).

#### 2.1.3 Emission factors

The Computer Programme calculating Emissions from Road Transport (COPERT) model is used to calculate emission factors for different vehicle types (38, 39). The relevant parameters and sources that need to be input to the model are as follows: the numbers of different types of vehicles were obtained as shown above. Fuel quality parameters were derived from Chinese national and local fuel standards. The average driving speeds of different vehicle types is based on previous studies (2, 14, 40, 41). Meteorological data (such as humidity and temperature) were from the China Meteorological Yearbook.

### 2.2 Air quality simulation

In this study, a multiscale air quality model (community multiscale air quality modeling system, CMAQ) developed by the US National Environmental Protection Agency was used to evaluate the contribution of vehicle pollution sources to atmospheric PM_2.5_ concentrations in different regions based on multiscale and multi-industry emission inventory data in China (EMEIC^1^) (42). First, the vehicle emissions calculated above were used to replace the traffic source emissions in the model list, and then the environmental air quality standards were simulated for the pollutant emission lists of the whole industry in 2010, 2015 and 2020 to obtain the average PM_2.5_ concentration in the study area. Second, through sensitivity simulation, transport source emissions were removed from the emission inventory, and CMAQ was used to simulate the annual average PM_2.5_ concentration after removing transport source emissions. Finally, the contribution rate of traffic sources to atmospheric PM_2.5_ concentration was assessed by analyzing the difference between standard and sensitivity simulations. This contribution rate was then multiplied by the high-resolution atmospheric PM_2.5_ concentration obtained from satellite inversion to obtain the atmospheric PM_2.5_ concentration caused by traffic sources. Atmospheric PM_2.5_ concentration data retrieved by satellite in this study were derived from research results in the literature (42–47).

### 2.3 Health effects evaluation method

In this study, an epidemiology-based Poisson regression relative risk model was used to quantify changes in the impact of increasing PM_2.5_ concentration on population health. The relevant calculation method is shown in Formula 3:


(3) $$\Delta Y=Y_{0}\cdot 1-E^{-\beta \Delta C}\cdot Pop$$

where ∆*Y* is the health impact estimation of a pollutant concentration change, *Y_0_* is the baseline incidence of the health effect endpoint (relevant data are shown in Table 1), *Pop* refers to the number of exposed people. In this study, the number of resident populations in each city is taken as the number of exposed populations. ∆*C* is the change in PM_2.5_ concentration in μg/m^3^. *β* refers to the exposure-response relationship coefficient. There is no consensus on whether there is a threshold concentration for the health effects of PM_2.5_ pollution from vehicles. In this paper, we refer to a study at the county level in China and conclude that there is no threshold concentration of PM_2.5_ pollution affecting health in China (51).

**Table 1:** The baseline incidence of the health effect endpoint/‰.

Health effect endpoint		Eastern city	Central city	Western city	References
Premature death	Premature death	Mortality rate of each city			Ministry of Health of the People’s Republic of China (49), National Health and Family Planning Commission of the People’s Republic of China (50, 51)
Inpatients	Respiratory diseases	8.8	8.9	16.9	
	Cardiovascular diseases	14.2	16.6	14.6	
Outpatients	Internal medicine	Outpatient rate of internal medicine of each city			
	Pediatrics	Outpatient rate of pediatrics of each city			
Illness	Acute bronchitis	28.1	24.9	53.2	
	Chronic bronchitis	4.6	4	10.3	

The health effect endpoints selected for this research were premature death, inpatients (respiratory and cardiovascular diseases), outpatients (internal medicine and pediatrics), and illness (acute bronchitis and chronic bronchitis). The exposure-response relationship coefficient (*β*) is one of the main parameters of health effect assessment, and the relevant data used in this study are shown in Table 2.

**Table 2:** Exposure-response relationship coefficients of the major health effect endpoints.

Health effect endpoint		*β*	95% CI	References
Premature death	Premature death	0.00296	0.00076–0.00504	Shang et al. (52)
Inpatients	Respiratory diseases	0.00109	0–0.00221	Chen et al. (54) and Wang (55)
	Cardiovascular diseases	0.00068	0.00043–0.00093	Chen et al. (54) and Wang (55)
Outpatients	Internal medicine	0.00049	0.00027–0.00070	Wang et al. (56)
	Pediatrics	0.00056	0.00020–0.00090	Wang et al. (56)
Illness	Acute bronchitis	0.0079	0.00270–0.01300	Wang et al. (56) and Huang et al. (57)
	Chronic bronchitis	0.01009	0.00366–0.01559	Wang et al. (56) and Huang et al. (57)

### 2.4 Monetization evaluation of health effects

In this research, the willingness to pay method and cost of illness analysis were used to estimate the health economic value of the death terminal and disease terminal, respectively. After solving the economic loss of each health terminal, all terminals are added. The unit cost of premature death was calculated using the value of statistical life (VSL) of Beijing residents in 2010 (57), which was 1.68 million yuan. The *per capita* disposable income of each city and the CPI index were converted into the corresponding values of each city in 2010, 2015 and 2020.

Inpatient loss was calculated using the cost of illness analysis, which calculates the unit cost of a certain disease *i* with the Formula (4) as follows:


(4) $$C_{i}=C_{ip}+GDP_{P}\times T_{iL}$$

where *C_i_* is the unit economic loss of health endpoint *i* caused by PM_2.5_ pollution (yuan); *C_ip_* is the medical cost per case of health endpoint *I* (yuan); *GDP_P_* is the cost of lost work per person per day [yuan·(person·d)^−1^]; and *T_iL_* refers to the delay time due to treatment health endpoint *i* (d).

The length of hospital stay *per capita* and direct costs *per capita* were derived from the corresponding yearbooks (48–50). In this study, the length of hospital days and direct costs *per capita* for cardiovascular diseases were replaced by the average value of major diseases such as acute myocardial infarction and congestive heart-failure. The length of hospital days for respiratory diseases and the direct costs *per capita* were replaced by the average value of major diseases such as invasive tuberculosis, bronchopneumonia, and bacterial pneumonia.

The cost of *per capita* per day of missing work was replaced by the daily average of annual *per capita* GDP, and the data on *per capita* GDP come from the relevant yearbooks [China City Statistical Yearbook (2011, 2016, 2021)] and are then converted into the daily average value. The delay time is the day of the inpatient stay.

The unit economic loss of outpatients (internal medicine and pediatrics) is calculated as follows: assuming that the proportion of all outpatient medical expenses in different cities in the study area is the same as the whole country, then the corresponding outpatient expenses of each city are calculated according to the *per capita* disposable income of the province and the city and the provincial outpatient medical expenses, and finally, the corresponding outpatient expenses of the internal medicine and pediatrics are obtained.

Chronic bronchitis has the characteristics of slow course, not easy to recover, and different to determine the duration of illness, often causes great suffering to patients and significantly reduces the quality of life, making it inappropriate to use cost of illness analysis to calculate the unit cost. According to the relevant literature (58), the unit economic loss of chronic bronchitis was 32% of the VSL. For the unit economic loss of acute bronchitis, assuming that the ratio of unit economic loss to outpatient loss is the same in each city, the loss of acute bronchitis in each city is calculated according to the unit loss ratio of outpatient and acute bronchitis by Huang et al. (56) and the research on the economic loss of outpatient units in the relevant literature (21, 53, 59, 60).

The unit economic losses of different health endpoints caused by PM_2.5_ pollution emitted from vehicles in each city of the three urban agglomerations are estimated based on the above methods. The unit economic losses of some cities are shown in Supplementary Table A3.

## 3 Results and discussion

### 3.1 Emission characteristics of PM_2.5_ from vehicles in three urban agglomerations

#### 3.1.1 Time variation trend of vehicle pollutants

**Figure 2:**
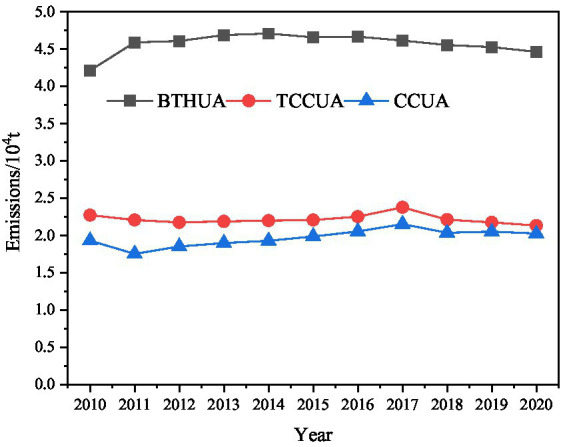
The variation trends of PM_2.5_ emissions from vehicles in the three urban agglomerations from 2010 to 2020.

On the basis of establishing vehicle pollutant emission inventory, the temporal variation trend of PM_2.5_ emissions from vehicles in the BTHUA, TCCUA and CCUA from 2010 to 2020 was analyzed (Figure 2), and there were differences in the temporal variation trend of pollutant emissions in different urban agglomerations.

PM_2.5_ emissions from vehicles in the BTHUA maintained a relatively stable growth trend from 2010 to 2014, increasing from 42,100 tons to 47,100 tons. However, PM_2.5_ emissions began to slowly decline in recent years, and pollutant emissions dropped to 44,600 tons by 2020. This was related to the early implementation of new vehicular emission standards in Beijing and Tianjin. In the TCCUA and CCUA, PM_2.5_ emissions from vehicles increased substantially, reaching 22,900 and 21,500 tons in 2017, up 9.24 and 22.82% compared with the lowest year, respectively. Although the total emission of PM_2.5_ of vehicle pollutants in the three major urban agglomerations fluctuated slightly in individual years, it was still high overall, and the emission reduction situation of vehicle pollutants was still very serious.

#### 3.1.2 Contribution rates by different vehicle types

**Figure 3:**
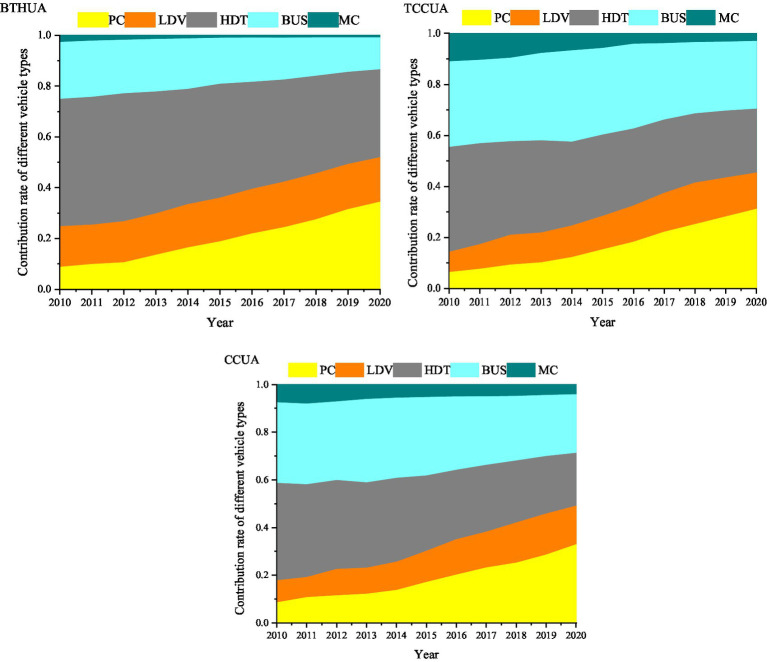
Contribution rate of different vehicles to total vehicular emissions in three urban agglomerations from 2010 to 2020.

The major contribution vehicles of PM_2.5_ pollutants in the BTHUA (2010–2014), TCCUA and CCUA were HDTs and BUSs from 2010 to 2020, and their contribution rates remained above 65.27, 51.55, and 46.73%, respectively (Figure 3). The reasons were chiefly as follows: (1) the fuel used by HDTs and BUSs was mainly diesel, which easily undergone incomplete combustion in the process of driving, resulting in the generation of particulate matter (61); and (2) HDTs and BUSs had higher average annual VKTs (31). Therefore, measures such as increasing efforts to eliminate high-polluting vehicles and promoting new-energy buses were crucial to effectively control pollutant emissions.

The emission sharing rate of different vehicle types in each urban agglomeration was analyzed. Due to the large population of HDTs in the BTHUA, HDTs were the largest contributor to the vehicle pollutant PM_2.5_. With the increasingly strict control of HDT driving in cities in recent years, the average annual VKTs of HDTs decreased, and the contribution rate of HDTs decreased to only 34.63% by 2020. In the TCCUA and CCUA, the largest contributor of PM_2.5_ from vehicles was also HDT. However, with the decrease in the average annual VKTs of HDTs in recent years, the proportion of BUSs increased to 26.54 and 24.58%, respectively, in 2020. In addition, the contribution rate of LDV to PM_2.5_ of vehicle pollutants in the three major urban agglomerations could not be ignored. In 2020, the contribution rates of LDV to PM_2.5_ in the three major urban agglomerations were 17.53, 14.21, and 16.21%, respectively. Due to the large increase in the number of LDVs, the contribution rate of LDVs to PM_2.5_ in the three urban agglomerations increased progressively year by year from 2010 to 2020, with an average annual growth rate of 14.83, 17.34, and 14.67%, respectively.

#### 3.1.3 Emission characteristics of pollutants from vehicles in different cities

**Figure 4:**
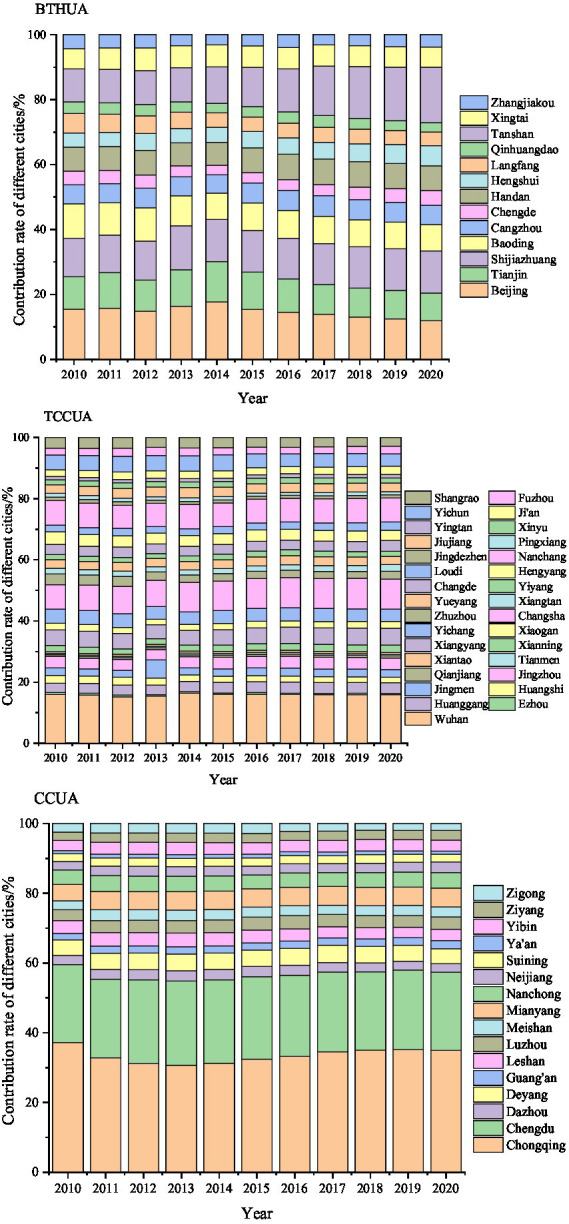
Contributions of different cities to total vehicular emissions in the three urban agglomerations from 2010 to 2020.

As shown in Figure 4, the cities with large vehicle pollutant emissions were Beijing, Tianjin, Shijiazhuang, and Tangshan in the BTHUA. Due to the large number of vehicles in Beijing, it accounted for a high proportion of the total emissions in the BTHUA. However, due to the early implementation of vehicle standards and fuel standards and the implementation of odd–even license plate and tail license plate restrictions, Beijing reduced the emission of vehicle pollutants, and the proportion of PM_2.5_ of vehicle pollutants decreased from 15.43% in 2010 to 11.87% in 2020. The major sources of PM_2.5_ from vehicles in Tianjin were HDTs and BUSs. Although the emissions of these two types of vehicles decreased significantly in recent years, their proportion remained above 8.51%. Due to the large increase in the vehicle population, the proportions of PM_2.5_ in Shijiazhuang and Tangshan of Hebei Province were on the rise, from 11.89 and 10.13% in 2010 to 12.99 and 17.05% in 2020, respectively. The proportions of other cities were small (accounting for less than 10%), but their proportions showed an upward trend. Therefore, the reduction of vehicle pollution in these cities should not be relaxed. Therefore, it was suggested that vehicle emission management in the BTH urban agglomeration should focus on cities in Hebei Province, such as improving vehicle emission standards, strengthening vehicle emission management, controlling total vehicle pollutant emissions, and implementing vehicle driving restrictions when necessary.

Due to the large number of cities (31) in the TCCUA, the contribution rate of each city was not as large as that of other urban agglomerations. However, in general, from 2010 to 2020, the contribution rates of Wuhan and Changsha were relatively large, while those of other cities were small. Pollutant emissions in Wuhan accounted for a relatively high contribution rate of total emissions in the TCCUA. From 2010 to 2020, the contribution rate of Wuhan remained above 15.23%. As the population of HDTs increased year by year, the contribution rate showed a fluctuating upward trend. As the population of vehicles in Changsha increased year by year, the contribution rate of Changsha increased from 7.91% in 2010 to 9.82% in 2020. The population of HDTs and BUSs in Nanchang was large and increasing year by year, so the emission of pollutant PM_2.5_ from vehicles could not be ignored. The contribution rates of other cities were relatively small, and the change trend was gentle. However, with the increasing in the population of vehicles year by year, its emissions may increase in the future. Therefore, the emission reduction of vehicle pollutants in these cities should not be relaxed.

As a western urban agglomeration, the number of vehicles in the CCUA increased substantially in recent years. In addition, its complex terrain was mainly mountainous, hilly and basin, which caused large emission factors of pollutants and was poor dispersion of air pollutants. Therefore, air pollution problems caused by pollutants produced by vehicles had attracted increasing attention. In the CCUA, the major contributing cities of vehicle pollutants were Chongqing and Chengdu. From 2010 to 2020, the contribution rates of these two cities remained above 54.82%. The contribution rate in Chongqing decreased first and then increased, increasing from 30.66% in 2013 to 34.95% in 2020. For Chengdu, another major contributor of vehicle pollutants in the CCUA, the contribution rate showed a downward trend, from the highest value of 24.16% in 2013 to 22.42% in 2020. The contribution rates in other cities were relatively small.

### 3.2 Health effects and health economic losses of PM_2.5_ pollution from vehicles

#### 3.2.1 Results of health risk assessment for residents

Due to the different values of PM_2.5_ concentration, exposed population, inpatient, incidence of illness and mortality in different cities, the impact of PM_2.5_ pollution on the health endpoint of different cities was also different. Similarly, the health effect of the same city in different years was also different. In 2010, 2015 and 2020, the overall health endpoint effects of PM_2.5_ pollution from vehicles in some cities showed the trend of fluctuation rising at first and then slow declining (Supplementary Table A4). The order of premature death and the total number of health terminals were similar in some cities in different years, but there were differences in the total number of health terminals. The largest total number of people related to different health endpoints in 2020 were Chongqing, followed by Beijing, Chengdu and Tianjin (Table 3). On the one hand, vehicle emissions accounted for a relatively high proportion in these cities, and the exposed population was large and densely distributed, among which the exposed population in Chongqing was significantly higher than that in other cities. On the other hand, it was also closely related to the high degree of population aging in these cities, and the degree of population aging in some cities were relatively serious. The older adult population had poor tolerance to PM_2.5_ pollution, and the probability of death or illness was higher (62). When the concentration of PM_2.5_ was high, the exposed population base was large, the aging problem was serious, and the health risk was great. Therefore, it is necessary to strengthen the control of air pollution in high-population density areas in the future and reduce the number of people actually exposed to pollutants. In recent years, the Chinese government has actively carried out pollution prevention and control, the emission of PM_2.5_ from vehicles had shown a slow decline, and the number of healthy terminal people related to vehicle particulate pollution had also shown a downward trend in some cities. Therefore, it is necessary to continue to increase the emission reduction of vehicle pollution in the future.

**Table 3:** Health risk and ranking of PM_2.5_ pollution from vehicles in some cities in 2020 (mean and 95% CI)/(person).

Cities	Premature death/10^3^	Inpatient		Outpatient		Illness		Ranking
		Respiratory diseases/10^3^	Cardiovascular diseases/10^3^	Internal medicine/10^4^	Pediatrics/10^4^	Acute bronchitis/10^4^	Chronic bronchitis/10^4^	
Beijing	14.20 (3.78, 23.35)	11.90 (0, 23.67)	7.17 (4.55, 9.77)	8.15 (4.51, 11.61)	3.02 (1.09, 4.83)	14.61 (5.44, 22.14)	2.95 (1.19, 4.17)	2
Tianjin	3.25 (0.85, 5.47)	2.67 (0, 5.38)	1.60 (1.01, 2.19)	2.54 (1.40, 3.62)	0.59 (0.21, 0.95)	3.53 (1.24, 5.63)	0.73 (0.27, 1.09)	4
Shijiazhuang	2.64 (0.69, 4.44)	2.17 (0, 4.36)	1.30 (0.82, 1.78)	1.50 (0.83, 2.14)	0.67 (0.24, 1.08)	2.86 (1.01, 4.57)	0.59 (0.22, 0.88)	7
Baoding	3.19 (0.83, 5.36)	2.63 (0, 5.28)	1.58 (1.00, 2.15)	1.82 (1.00, 2.60)	0.82 (0.29, 1.31)	3.45 (1.22, 5.47)	0.71 (0.27, 1.06)	6
Handan	2.25 (0.59, 3.78)	1.85 (0, 3.72)	1.11 (0.70, 1.51)	1.28 (0.71, 1.83)	0.57 (0.21, 0.92)	2.44 (0.86, 3.89)	0.50 (0.19, 0.75)	10
Tangshan	2.49 (0.65, 4.17)	2.05 (0, 4.12)	1.23 (0.78, 1.68)	1.42 (0.79, 2.03)	0.64 (0.23, 1.02)	2.67 (0.95, 4.22)	0.55 (0.21, 0.81)	8
Wuhan	3.63 (0.95, 6.08)	3.17 (0, 6.37)	2.10 (1.33, 2.86)	1.66 (0.91, 2.36)	1.01 (0.36, 1.63)	3.46 (1.230, 5.49)	0.70 (0.27, 1.04)	5
Changsha	2.6 (0.68, 4.39)	2.28 (0, 4.59)	1.51 (0.95, 2.06)	1.22 (0.67, 1.74)	0.80 (0.29, 1.28)	2.50 (0.89, 3.98)	0.51 (0.19, 0.75)	9
Nanchang	1.30 (0.34, 2.19)	1.13 (0, 2.28)	0.75 (0.47, 1.02)	0.78 (0.43, 1.11)	0.34 (0.12, 0.54)	1.25 (0.44, 2.01)	0.25 (0.10, 0.38)	15
Chongqing	13.71 (3.61, 22.82)	19.65 (0, 39.36)	7.02 (4.45, 9.58)	7.42 (4.05, 10.45)	3.42 (1.23, 5.48)	27.48 (9.94, 42.82)	6.64 (2.58, 9.67)	1
Chengdu	7.78 (2.04, 12.93)	11.11 (0, 22.29)	3.97 (2.52, 5.42)	3.91 (2.16, 5.57)	2.38 (0.86, 3.81)	15.69 (5.63, 24.63)	3.80 (1.47, 5.58)	3

From different health endpoints, the number of premature deaths in some cities were higher than the number of respiratory and cardiovascular inpatients but significantly lower than the number of outpatients. The number of inpatients for respiratory and cardiovascular diseases did not vary significantly between years. There were more cases of acute and chronic bronchitis, and the number of cases exceeded the number of premature deaths, inpatient visits and outpatient visits. In terms of the number of illnesses, the number of acute bronchitis cases were much higher than that of chronic bronchitis cases. Among them, acute bronchitis accounted for more than 80% of the total number of illnesses, accounting for more than 43% of the total health effects (Supplementary Table A5).

**Figure 5:**
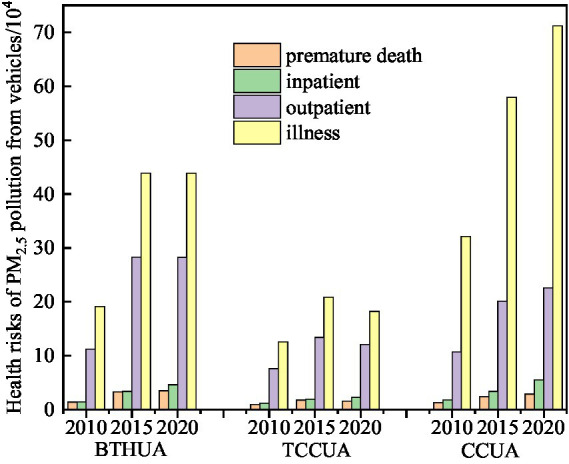
The change trend of health risks of PM_2.5_ pollution from vehicles for the three urban agglomerations.

Table 4 showed the number of each health endpoint and overall incidence caused by PM_2.5_ pollution from vehicles in different regions in 2010, 2015 and 2020. In the BTHUA and CCUA, the number of each health endpoint caused by PM_2.5_ pollution from vehicles showed an overall upward trend, while the TCCUA showed a downward trend in recent years (Figure 5), indicating that the emission reduction situation of vehicle pollution in the BTHUA and CCUA was not optimistic. Among them, the number of premature deaths and inpatients attributed to vehicle PM_2.5_ pollution in the BTHUA was higher than that in the TCCUA and CCUA, which was partly related to the more serious vehicle PM_2.5_ pollution in the BTHUA (Figure 2). The total incidence of health endpoints in the TCCUA was the highest, at 0.51% (95% CI: 0.19–0.80%), 0.91 (95% CI: 0.34–1.41%), and 0.99% (95% CI: 0.37–1.55%), respectively, and was related to the higher baseline incidence in the western urban agglomeration (Table 1). The risk of acute bronchitis and chronic bronchitis in western cities was higher than that in eastern and central cities, and the number of people suffering from acute bronchitis and chronic bronchitis in the CCUA was significantly higher than that in the other two urban agglomerations.

**Table 4:** Health risks of PM_2.5_ pollution from vehicles for the three urban agglomerations (mean and 95% CI).

Urban agglomeration	Years	Premature death/10^4^	Inpatient		Outpatient		Illness		Overall incidence of health endpoint/person·(10,000 people)^−1^
			Respiratory diseases/10^4^	Cardiovascular diseases/10^4^	Internal medicine/10^4^	Pediatrics/10^4^	Acute bronchitis/10^5^	Chronic bronchitis/10^4^	
BTHUA	2010	1.33 (0.35, 2.25)	0.70 (0, 1.42)	0.71 (0.45, 0.97)	7.77 (4.29, 11.09)	3.44 (1.23, 5.51)	1.58 (0.55, 2.55)	3.28 (1.22, 4.96)	32 (12, 49)
	2015	3.25 (0.85, 5.43)	1.66 (0, 3.34)	1.68 (1.06, 2.29)	20.25 (11.18, 28.66)	8.03 (2.88, 12.86)	3.64 (1.30, 5.75)	7.48 (2.86, 11.07)	71 (29, 108)
	2020	3.43 (0.90, 5.72)	2.84 (0, 5.70)	1.71 (1.08, 2.33)	20.30 (11.21, 28.94)	7.93 (2.84, 12.70)	3.64 (1.31, 5.70)	7.45 (2.89, 10.92)	73 (29, 112)
TCCUA	2010	0.94 (0.24, 1.60)	0.53 (0, 1.06)	0.61 (0.39, 0.84)	4.98 (2.74, 7.11)	2.58 (0.92, 4.15)	1.04 (0.36, 1.70)	2.13 (0.79, 3.24)	17 (7, 27)
	2015	1.70 (0.44, 2.86)	0.88 (0, 1.78)	1.03 (0.65, 1.40)	8.60 (4.74, 12.28)	4.75 (1.70, 7.62)	1.73 (0.61, 2.79)	3.52 (1.32, 5.31)	29 (12, 46)
	2020	1.56 (0.40, 2.63)	1.35 (0, 2.73)	0.89 (0.57, 1.22)	7.68 (4.24, 10.97)	4.39 (1.57, 7.04)	1.51 (0.53, 2.43)	3.06 (1.14, 4.62)	27 (10, 42)
CCUA	2010	1.25 (0.32, 2.12)	1.16 (0, 2.34)	0.63 (0.40, 0.86)	6.99 (3.85, 9.97)	3.70 (1.32, 5.94)	2.58 (0.90, 4.16)	6.32 (2.35, 9.56)	51 (19, 80)
	2015	2.38 (0.62, 3.99)	2.15 (0, 4.33)	1.16 (0.74, 1.59)	13.22 (7.30, 18.86)	6.86 (2.38, 10.64)	4.66 (1.66, 7.37)	11.32 (4.32, 16.79)	91 (34, 141)
	2020	2.83 (0.74, 4.73)	4.04 (0, 8.11)	1.44 (0.91, 1.97)	14.70 (8.07, 20.83)	7.84 (2.81, 12.57)	5.73 (2.05, 9.02)	13.90 (5.33, 20.50)	99 (37, 155)

#### 3.2.2 Results of health economic loss for residents

In 2010, 2015 and 2020, the health and economic losses, the proportion of health economic loss to GDP and the *per capita* economic losses caused by PM_2.5_ pollution from vehicles in most cities of the three major urban agglomerations showed a fluctuating trend, while other cities showed a slow downward trend in recent years (Supplementary Table A5). Among them, the top 10 cities were Beijing, Chongqing, Chengdu, Wuhan, Tianjin, Changsha, Tangshan, Shijiazhuang, Baoding and Nanchang, ranked by the value of health economic loss of residents in 2020 (Table 5). However, the top 10 cities ranked by the proportion of health economic loss to GDP were Chongqing, Beijing, Chengdu, Baoding, Nanchong, Tangshan, Mianyang, Xingtai, Handan and Deyang. Ranking by health economic loss *per capita*, Beijing was the largest, followed by Chongqing, Chengdu, Wuhan and Tangshan. In general, the health economic losses caused by vehicle PM_2.5_ pollution were relatively serious in Beijing, Chengdu, Chongqing, Tianjin, Wuhan and Tangshan.

**Table 5:** Health economic losses of PM_2.5_ pollution from vehicles in major cities in 2020 (mean and 95% CI).

Cities	Health economic loss/100 million yuan	The proportion of health economic loss to GDP/%	Health economic loss *per capita*/yuan	Ranked by health economic loss	Ranked by the proportion of health economic loss to GDP	Ranking by health economic loss *per capita*
Beijing	805.83 (253.67, 1263.14)	2.24 (0.70, 3.51)	3680.75 (1158.70, 5769.61)	1	2	1
Tianjin	127.36 (39.97, 203.52)	0.88 (0.28, 1.40)	918.36 (288.19, 1467.57)	5	16	7
Shijiazhuang	57.20 (17.61, 92.00)	0.96 (0.29, 1.55)	508.82 (156.62, 818.39)	8	12	14
Baoding	55.83 (17.69, 88.72)	1.41 (0.45, 2.24)	483.57 (153.25, 768.41)	9	4	15
Cangzhou	34.28 (10.75, 54.86)	0.93 (0.29, 1.48)	469.48 (147.27, 751.27)	13	14	17
Handan	38.69 (12.26, 61.57)	1.06 (0.33, 1.69)	410.92 (130.25, 653.96)	11	9	16
Tangshan	86.56 (27.09, 137.79)	1.21 (0.38, 1.92)	1121.56 (351.06, 1785.36)	7	6	5
Wuhan	154.79 (47.57, 248.21)	0.99 (0.30, 1.59)	1243.58 (382.13, 1994.03)	4	11	4
Changsha	106.26 (32.55, 170.82)	0.88 (0.27, 1.41)	1056.19 (323.55, 1697.84)	6	17	6
Nanchang	42.32 (12.91, 68.29)	0.74 (0.22, 1.19)	676.51 (206.40, 1091.83)	10	26	10
Chongqing	615.10 (208.85, 947.37)	2.41 (0.82, 3.71)	1916.86 (650.85, 2952.31)	2	1	2
Chendu	376.09 (126.84, 582.34)	2.12 (0.72, 3.29)	1795.47 (605.51, 2780.06)	3	3	3

The total health economic losses caused by PM_2.5_ pollution from vehicles in the three major urban agglomerations in 2010, 2015 and 2020 were 68.25 billion yuan (95% CI: 21.65–109.16), 206.33 billion yuan (95% CI: 66.20–326.20) and 300.73 billion yuan (95% CI: 96.79–473.16), accounting for 0.67% (95% CI: 0.21–1.07%), 1.19% (95% CI: 0.38–1.88%) and 1.21% (95% CI: 0.39–1.90%) of the total GDP of these cities and accounting for 0.17% (95% CI: 0.05–0.26%), 0.30% (95% CI: 0.09–0.47%) and 0.30% (95% CI: 0.09–0.47%) of national GDP, respectively. Among them, the health economic losses of the top five cities accounted for 60.44, 59.87 and 69.14% of all health economic losses.

**Figure 6:**
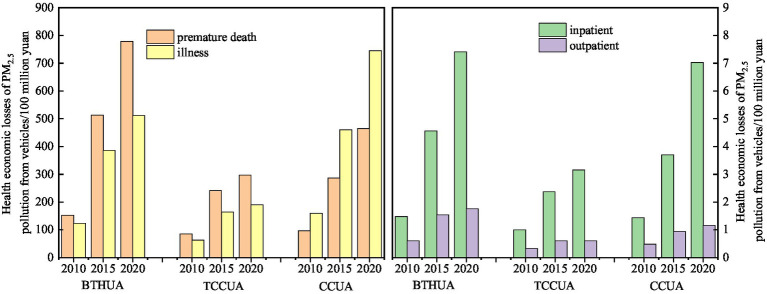
The change trend of health economic losses of PM_2.5_ pollution from vehicles for three urban agglomerations.

The health economic loss and health economic loss *per capita* caused by PM_2.5_ pollution from vehicles in the BTHUA, TCCUA, and CCUA showed an upward trend overall (Figure 6), and the proportion of health economic loss to GDP decreased in some years (Table 6). Compared to 2010, the health economic loss in BTH increased by 102.22 billion yuan (95% CI: 32.21–160.60 billion yuan), and the health economic loss *per capita* increased by 911.84 yuan (95% CI: 287.41–1,432.04 yuan) in 2020. The growth trend of the proportion of health economic loss to GDP in 2020 slowed, but it still increased by 0.87% (95% CI: 0.27–1.35%) compared with 2010. Health economic losses and health economic losses *per capita* in the TCCUA and CCUA also showed an upward trend compared with the BTHUA, but the proportion of health economic losses to GDP showed a downward trend in 2020, but it was still higher than that in 2010, and the proportion of health economic losses to GDP in 2020 increased by 0.1% (95% CI: 0.03–0.16%) and 0.66% (95% CI: 0.22–0.99%) compared with 2010, respectively. Premature death and chronic bronchitis were the most important health losses caused by PM_2.5_ pollution from vehicles, accounting for more than 96.84%. The proportion of health economic losses caused by premature death in the BTHUA and the TCCUA were the largest, reaching more than 45.85 and 57.21%, respectively, followed by chronic bronchitis, accounting for more than 35.55 and 37.93% of the total losses. The proportion of health economic loss caused by chronic bronchitis in the CCUA was the highest, reaching more than 59.83%, followed by premature death, acute bronchitis, cardiovascular disease hospitalization, respiratory disease hospitalization, internal medicine and pediatrics.

**Table 6:** Health economic losses of PM_2.5_ pollution from vehicles for three urban agglomerations (mean and 95% CI).

	Years	Health economic loss/100 million yuan						
		Premature death/10^2^	Inpatient		Outpatient		Illness	
			Respiratory diseases	Cardiovascular diseases	Internal medicine	Pediatrics	Acute bronchitis	Chronic bronchitis/10^2^
BTHUA	2010	1.52 (0.40, 2.57)	0.48 (0, 0.97)	0.99 (0.63, 1.36)	0.43 (0.23, 0.61)	0.18 (0.06, 0.29)	3.42 (1.19, 5.50)	1.20 (0.45, 1.80)
	2015	5.13 (1.34, 8.59)	1.41 (0, 2.84)	3.15 (1.99, 4.29)	1.13 (0.62, 1.59)	0.41 (0.15, 0.66)	9.04 (3.23, 14.27)	3.77 (1.45, 5.58)
	2020	7.78 (2.06, 12.89)	3.17 (0, 6.36)	4.24 (2.69, 5.78)	1.29 (0.71, 1.84)	0.47 (0.17, 0.76)	12.04 (4.34, 18.86)	5.00 (1.95, 7.30)
TCCUA	2010	0.85 (0.22, 1.44)	0.29 (0, 0.59)	0.71 (0.45, 0.97)	0.22 (0.12, 0.31)	0.11 (0.04, 0.18)	1.41 (0.49, 2.28)	0.61 (0.23, 0.92)
	2015	2.41 (0.63, 4.05)	0.69 (0, 1.40)	1.68 (1.06, 2.29)	0.40 (0.22, 0.57)	0.21 (0.08, 0.34)	3.02 (1.06, 4.85)	1.61 (0.60, 2.41)
	2020	2.97 (0.77, 5.00)	1.38 (0, 2.79)	1.77 (1.12, 2.42)	0.39 (0.22, 0.56)	0.22 (0.08, 0.35)	3.77 (1.32, 6.06)	1.86 (0.70, 2.80)
CCUA	2010	0.96 (0.25, 1.62)	0.66 (0.1.34)	0.77 (0.49, 1.05)	0.32 (0.18, 0.45)	0.16 (0.06, 0.25)	4.23 (1.48, 6.81)	1.55 (0.58, 2.34)
	2015	2.86 (0.75, 4.79)	1.75 (0, 3.51)	1.95 (1.23, 2.66)	0.63 (0.35, 0.90)	0.31 (0.11, 0.48)	9.73 (3.47, 15.35)	4.50 (1.72, 6.64)
	2020	4.64 (1.22, 7.76)	4.12 (0, 8.28)	2.90 (1.84, 3.96)	0.78 (0.43, 1.10)	0.38 (0.14, 0.61)	16.42 (5.88, 25.80)	7.28 (2.80, 10.72)

	Years	The proportion of health economic loss to GDP/%	Health economic loss *per capita*/10^2^ yuan
BTHUA	2010	0.63 (0.20, 1.02)	2.65 (0.82, 4.27)
	2015	1.44 (0.45, 2.29)	8.13 (2.56, 12.93)
	2020	1.48 (0.47, 2.36)	11.77 (3.40, 18.59)
TCCUA	2010	0.42 (0.13, 0.68)	1.17 (0.36, 1.90)
	2015	0.61 (0.19, 0.99)	3.18 (0.98, 5.12)
	2020	0.52 (0.16, 0.84)	3.87 (1.18, 6.26)
CCUA	2010	1.11 (0.37, 1.75)	2.68 (0.88, 4.23)
	2015	1.71 (0.58, 2.66)	7.63 (2.57, 11.87)
	2020	1.77 (0.59, 2.74)	11.85 (3.99, 18.37)

## 4 Uncertainty evaluation

This study estimated PM_2.5_ emissions from vehicles in the BTHUA, TCCUA and CCUA from 2010 to 2020, analyzed their emission characteristics, and assessed the health effects and economic losses caused by PM_2.5_ pollution. However, there are inevitable statistical errors, model design errors and systematic errors in the process of establishing a pollutant discharge inventory and health effects and economic loss assessment, so it is necessary to analyze and discuss the uncertainty factors in the assessment process.

In this study, the uncertainties of vehicle emission estimation mainly come from the following aspects. First, the data sources of the vehicle population came from national statistical yearbooks, local statistical yearbooks and related studies. The time limitations of panel data can cause errors in inventory estimation. Second, regarding the activity level data of vehicles, the average annual VKTs in this study used only the statistical data of some typical cities and referred to the relevant literatures, which led to certain uncertainties in the average annual VKTs. Therefore, increasingly extensive data testing and investigation should be carried out during the research process to minimize the uncertainty caused by the average annual VKTs. Third, regarding emission factor data, although the COPERT model has been widely used, the error between the model simulation value and the actual emission value can bring uncertainty to the emission factor.

Regarding the health effects and economic losses caused by pollution, this paper considered only the health effects caused by PM_2.5_ and did not consider other pollutants, such as PM_10_, O_3_, SO_2_, and NO_x_. Due to the potential synergistic effects of various contaminants on health when they enter the human body, this assessment may be limited. The population data came from the permanent population data of each city. Since the total population of each city is always in the process of dynamic change, this study did not consider the dynamic of population and selected the permanent population for estimation, which has certain errors.

Due to the lack of basic health data, the health endpoints assessed were not sufficiently comprehensive. This study analyzed only premature deaths, inpatient visits (respiratory and cardiovascular diseases), outpatient visits (internal medicine and pediatrics), and illnesses (acute bronchitis and chronic bronchitis) and did not consider reduced lung function or adverse reproduction. Cost of illness analysis was used to estimate the unit economic loss of the health endpoints of related diseases. In the calculation process of this study, only medical costs and lost work costs were considered, and self-treatment expenses and traveling expenses were not considered. Although the WTP method is superior to the above methods in this respect, it also ignores the economic investment of the public sector in medical treatment, medical research and development in the calculation process, so there is a certain deviation from the actual situation. At the same time, evaluation indicators such as VSL and CPI in monetization also have a strong correlation with local economic development conditions, and it is best to use localized data. In this study, the local VSL research results of Beijing are used in the calculation, and the VSL value of each city is calculated according to the loss of exposed population in Beijing and the *per capita* disposable income of each city to improve the accuracy as much as possible.

## 5 Conclusion

From 2010 to 2020, the emission characteristics of vehicle pollutants in the three major urban agglomerations were different. The emission reduction trend from vehicles in the BTHUA was better than that in the TCCUA and the CCUA in recent years. The PM_2.5_ emissions of the TCCUA and the CCUA showed an obvious upward trend from 2010 to 2017. However, PM_2.5_ emissions in the BTHUA showed a significant downward trend in recent years, indicating that the promulgation and implementation of reasonable and effective emission reduction measures in advance is very important for pollutant reduction.

From 2010 to 2020, the major contributing vehicles of PM_2.5_ pollutants in the BTHUA (2010–2014), TCCUA and CCUA were HDTs and BUSs. Therefore, it is necessary to formulate corresponding emission reduction measures for the main contributing vehicles. In addition, the emission characteristics of vehicle pollutants in different urban agglomerations were significantly different. The contribution rate of Beijing in the BTHUA decreased significantly. In addition to the capital cities, the growth trend of pollutants in other cities cannot be ignored, and corresponding measures should be taken to promote the emission reduction of pollutants according to the situation and characteristics of each city.

In the BTHUA and CCUA, the changes in the number of health endpoints caused by PM_2.5_ pollution from vehicles showed an overall upward trend in 2010, 2015 and 2020, while those in the TCCUA showed a downward trend in recent years. Among them, the number of premature deaths, inpatients and outpatients attributed to PM_2.5_ pollution from vehicles in the BTHUA was higher than that in the TCCUA and the CCUA, while the number of people suffering from acute bronchitis and chronic bronchitis was significantly higher than that in the other two urban agglomerations due to the higher baseline incidence in the CCUA. Chongqing had the largest number of people who experienced the health endpoints, followed by Beijing and Chengdu. Therefore, it is necessary to strengthen the control of air pollution in high-population density areas in the future and reduce the number of people actually exposed to pollutants.

In 2010, 2015 and 2020, the health economic losses and *per capita* economic losses caused by PM_2.5_ pollution from vehicles in the three major urban agglomerations showed an overall upward trend, while the proportion of economic loss in GDP decreased in some years. The health and economic losses caused by PM_2.5_ pollution from vehicles in different cities were also different, but health economic losses were relatively prominent in Beijing, Chengdu, Chongqing, Tianjin, and Wuhan.

## Data availability statement

The original contributions presented in the study are included in the article/Supplementary material, further inquiries can be directed to the corresponding author.

## Author contributions

XS: Writing – review & editing, Writing – original draft, Methodology. YH: Writing – review & editing, Software.

## Footnotes

^1^http://www.meicmodel.org
