# Supplementary material for: Emission characteristics and health effects of PM2.5 from vehicles in typical areas
Source: Front Public Health. 2024 Jun 19;12:1326659. doi: 10.3389/fpubh.2024.1326659 (PMC11220272; doi:10.3389/fpubh.2024.1326659)
Supplement: Supplementary file 1 [file Data_Sheet_1.docx]

Supplementary Table A1 Vehicular emission standards implementation timetable

| Cities | Vehicle types | State I | State II | State III | State IV | State Ⅴ | State Ⅵ |
| --- | --- | --- | --- | --- | --- | --- | --- |
| Beijing | PC, LDV | 1999 | 2003 | 2006 | 2008 | 2013 | 2017 |
|  | BUS, HDT | 2000 | 2003 | 2006 | 2008 | 2015 | 2020 |
|  | MC | 2002 | 2004 | 2008 | / | / | / |
| Tianjin | PC, LDV | 2000/2001 | 2004/2006 | 2008 | 2012 | 2015 | 2017 |
|  | BUS, HDT | 2001 | 2005 | 2008 | 2013 | 2016 | 2019 |
|  | MC | 2002 | 2004 | 2010 | / | / | / |
| Other cities | PC, LDV | 2000/2001 | 2004/2006 | 2008 | 2012 | 2017 | 2018 |
|  | BUS, HDT | 2001 | 2005 | 2008 | 2015 | 2017 | / |
|  | MC | 2002 | 2004 | 2010 | / | / | / |

Supplementary Table A2 The survival rates of vehicles

| Vehicle age | PC | LDV | HDT | BUS | MC | Vehicle age | PC | LDV | HDT | BUS | MC |
| --- | --- | --- | --- | --- | --- | --- | --- | --- | --- | --- | --- |
| 1 | 1.00 | 1.00 | 1.00 | 1.00 | 1.00 | 11 | 0.80 | 0.01 | 0.65 | 0.45 | 0.00 |
| 2 | 1.00 | 1.00 | 1.00 | 1.00 | 0.90 | 12 | 0.72 | 0.00 | 0.50 | 0.28 | 0.00 |
| 3 | 1.00 | 0.99 | 1.00 | 0.99 | 0.80 | 13 | 0.61 | 0.00 | 0.34 | 0.15 | 0.00 |
| 4 | 0.99 | 0.99 | 0.99 | 0.99 | 0.70 | 14 | 0.47 | 0.00 | 0.17 | 0.07 | 0.00 |
| 5 | 0.98 | 0.93 | 0.98 | 0.98 | 0.60 | 15 | 0.33 | 0.00 | 0.07 | 0.03 | 0.00 |
| 6 | 0.98 | 0.79 | 0.98 | 0.95 | 0.50 | 16 | 0.20 | 0.00 | 0.03 | 0.02 | 0.00 |
| 7 | 0.97 | 0.58 | 0.95 | 0.92 | 0.40 | 17 | 0.10 | 0.00 | 0.01 | 0.00 | 0.00 |
| 8 | 0.95 | 0.32 | 0.92 | 0.85 | 0.30 | 18 | 0.04 | 0.00 | 0.01 | 0.00 | 0.00 |
| 9 | 0.92 | 0.10 | 0.85 | 0.75 | 0.20 | 19 | 0.02 | 0.00 | 0.00 | 0.00 | 0.00 |
| 10 | 0.87 | 0.03 | 0.78 | 0.61 | 0.10 | 20 | 0.00 | 0.00 | 0.00 | 0.00 | 0.00 |

Supplementary Table A3 The unit economic losses of different health endpoints of some cities in the three urban agglomerations in 2010, 2015, and 2020

| Cities | premature death | | | inpatient | | | | | | outpatient | | | | | | illness | | | | | |
| --- | --- | --- | --- | --- | --- | --- | --- | --- | --- | --- | --- | --- | --- | --- | --- | --- | --- | --- | --- | --- | --- |
|  | VSL/ 10 thousand yuan·person^-1^ | | | Respiratory diseases / yuan·person^-1^ | | | cardiovascular diseases / yuan·person^-1^ | | | internal medicine / yuan·person^-1^ | | | pediatrics / yuan·person^-1^ | | | acute bronchitis / yuan·person^-1^ | | | chronic bronchitis / 10 thousand yuan·person^-1^ | | |
|  | 2010 | 2015 | 2020 | 2010 | 2015 | 2020 | 2010 | 2015 | 2020 | 2010 | 2015 | 2020 | 2010 | 2015 | 2020 | 2010 | 2015 | 2020 | 2010 | 2015 | 2020 |
| Beijing | 168 | 275.5 | 357.1 | 8754 | 10863 | 14542 | 20214 | 27541 | 34521 | 742 | 773 | 795 | 710 | 725 | 763 | 3250 | 3748 | 4321 | 53.8 | 88.2 | 114.3 |
| Tianjin | 162.8 | 222.6 | 211.2 | 7410 | 9074 | 12014 | 14641 | 19811 | 25541 | 575 | 613 | 643 | 543 | 581 | 612 | 2541 | 2972 | 3654 | 52.1 | 71.2 | 67.6 |
| Shijiazhuang | 88.2 | 122.3 | 127.2 | 6237 | 8120 | 9854 | 10933 | 15505 | 19874 | 450 | 480 | 520 | 435 | 460 | 500 | 1650 | 1985 | 2700 | 28.2 | 39.1 | 40.7 |
| Baoding | 54.2 | 77.9 | 93.1 | 5676 | 6939 | 8541 | 10869 | 14796 | 18541 | 450 | 480 | 520 | 435 | 460 | 500 | 1630 | 1980 | 2700 | 17.3 | 24.9 | 29.8 |
| Cangzhou | 82.2 | 110.2 | 121.7 | 6068 | 8095 | 9421 | 10492 | 15417 | 19774 | 440 | 470 | 510 | 427 | 445 | 480 | 1800 | 2300 | 2800 | 26.3 | 35.3 | 38.9 |
| Handan | 71.6 | 87.2 | 89.7 | 5372 | 7207 | 7954 | 10550 | 15075 | 17041 | 415 | 437 | 476 | 405 | 420 | 450 | 1300 | 1700 | 2350 | 22.9 | 27.9 | 28.7 |
| Tangshan | 138 | 172.3 | 198.5 | 6402 | 8337 | 9541 | 11632 | 16252 | 18854 | 450 | 480 | 525 | 435 | 460 | 495 | 1900 | 2300 | 2950 | 44.2 | 55.1 | 63.5 |
| Wuhan | 137.2 | 216.3 | 260.6 | 6571 | 8830 | 12354 | 14180 | 18661 | 23351 | 550 | 584 | 604 | 530 | 550 | 585 | 1900 | 2300 | 2985 | 43.9 | 69.2 | 83.4 |
| Changsha | 150.9 | 234.9 | 247.6 | 7191 | 9826 | 13254 | 14411 | 18970 | 20443 | 540 | 575 | 590 | 520 | 540 | 575 | 1800 | 2200 | 2900 | 48.3 | 75.2 | 79.2 |
| Nanchang | 108.1 | 167.9 | 197.1 | 6032 | 8889 | 11245 | 13718 | 17889 | 18748 | 450 | 480 | 520 | 435 | 460 | 500 | 1600 | 2000 | 2750 | 34.6 | 53.7 | 63.1 |
| Chongqing | 74.7 | 124.7 | 172.3 | 6055 | 8378 | 10585 | 13218 | 17283 | 18641 | 480 | 504 | 554 | 445 | 468 | 505 | 2043 | 2443 | 3250 | 23.9 | 39.9 | 55.1 |
| Chengdu | 117.3 | 165.0 | 185 | 6452 | 8947 | 11597 | 13635 | 17846 | 19373 | 505 | 535 | 570 | 460 | 485 | 525 | 1800 | 2200 | 2950 | 37.6 | 52.8 | 59.2 |

Supplementary Table A4 Health risk of PM_2.5_ pollution from vehicles in the three urban agglomerations (mean and 95% CI) (/person)

| Cities | Years | premature death | inpatient | | outpatient | | illness | |
| --- | --- | --- | --- | --- | --- | --- | --- | --- |
|  |  |  | Respiratory diseases | cardiovascular diseases | internal medicine | pediatrics | acute bronchitis | chronic bronchitis |
| Beijing | 2010 | 3977(1034,6693) | 2097(0,4225) | 2116(1340,2889) | 23999(13240,34244) | 10903(3902,17489) | 46725(16437,74764) | 9652(3628,14471) |
|  | 2015 | 7841(2057,13086) | 4029(0,8080) | 4072(2581,5556) | 47759(26373,65904) | 17331(6211,27760) | 87321(31373,136935) | 17882(6897,26241) |
|  | 2020 | 14198(3784,23346) | 11897(0,23667) | 7171(4554,9766) | 81525(45091,116047) | 30214(10858,48277) | 146096(54444,221419) | 29476(11887,41729) |
| Tianjin | 2010 | 1547(400,2616) | 812(0,1641) | 819(518,1119) | 10699(5899,15274) | 3377(1207,5421) | 18383(6390,29756) | 3816(1414,5793) |
|  | 2015 | 3697(963,6212) | 1888(0,3800) | 1905(1207,2602) | 30840(17017,43998) | 5076(1817,8139) | 41839(14778,66685) | 8628(3259,12882) |
|  | 2020 | 3252(846,5468) | 2669(0,5376) | 1602(1014,2187) | 25404(14017,36246) | 5939(2126,9527) | 35274(12434,56331) | 7280(2743,10892) |
| Shijiazhuang | 2010 | 1395(361,2357) | 733(0,1480) | 739(468,1010) | 7678(4234,10960) | 3585(1281,5753) | 16536(5763,26699) | 3429(1274,5192) |
|  | 2015 | 3175(829,5319) | 1626(0,3268) | 1642(1041,2242) | 18797(10375,26808) | 8781(3145,14073) | 35664(12699,56409) | 7330(2797,10856) |
|  | 2020 | 2638(686,4436) | 2166(0,4362) | 1299(823,1775) | 14999(8276,21401) | 6735(2411,10802) | 28617(10088,45699) | 5906(2226,8836) |
| Baoding | 2010 | 1599(414,2702) | 841(0,1697) | 848(537,1158) | 8808(4858,12572) | 4112(1471,6599) | 18948(6608,30570) | 3928(1461,5942) |
|  | 2015 | 4238(1112,7070) | 2178(0,4368) | 2202(1396,3004) | 25207(13919,35935) | 11773(4219,18858) | 47159(16957,73898) | 9654(3727,14156) |
|  | 2020 | 3193(833,5357) | 2626(0,5283) | 1577(999,2152) | 18202(10045,25964) | 8172(2926,13102) | 34452(12212,54724) | 7094(2691,10554) |
| Handan | 2010 | 1094(283,1851) | 574(0,1160) | 579(366,791) | 6016(3317,8588) | 2809(1004,4509) | 13003(4520,21047) | 2699(1000,4098) |
|  | 2015 | 3065(802,5126) | 1572(0,3156) | 1588(1006,2167) | 18179(10037,25922) | 8492(3042,13607) | 34298(12260,54045) | 7038(2698,10381) |
|  | 2020 | 2251(586,3784) | 1848(0,3721) | 1109(702,1514) | 12801(7063,18264) | 5748(2057,9218) | 24403(8607,38948) | 5035(1899,7529) |
| Tangshan | 2010 | 918(238,1553) | 482(0,974) | 486(308,664) | 5050(2785,7209) | 2358(843,3785) | 10911(3794,17656) | 2265(839,3437) |
|  | 2015 | 2494(652,4172) | 1279(0,2568) | 1292(819,1763) | 14787(8163,21086) | 6907(2475,11068) | 27927(9976,44037) | 5733(2196,8462) |
|  | 2020 | 2489(651,4166) | 2052(0,4122) | 1232(781,1682) | 14231(7856,20294) | 6389(2289,10239) | 26700(9523,42165) | 5484(2096,8108) |
| Wuhan | 2010 | 1977(513,3331) | 1053(0,2123) | 1227(777,1677) | 10084(5563,14391) | 5575(1994,8944) | 20647(7238,33147) | 4191(1569,6307) |
|  | 2015 | 3192(834,5345) | 1653(0,3322) | 1930(1223,2635) | 14955(8255,21328) | 8234(2949,13197) | 31748(11311,50187) | 6402(2444,9475) |
|  | 2020 | 3625(946,6078) | 3166(0,6366) | 2095(1327,2860) | 16555(9137,23614) | 10142(3632,16258) | 34603(12290,54862) | 6986(2657,10373) |
| Changsha | 2010 | 1050(272,1773) | 559(0,1127) | 651(412,889) | 5234(2987,7472) | 2772(992,4449) | 11015(3845,17756) | 2240(834,3385) |
|  | 2015 | 1966(513,3299) | 1017(0,2045) | 1186(752,1620) | 9787(5402,13961) | 6120(2191,9812) | 19650(6965,31213) | 3971(1507,5907) |
|  | 2020 | 2612(681,4387) | 2278(0,4594) | 1506(954,2057) | 12218(6742,17430) | 7973(2854,12785) | 25031(8853,39845) | 5063(1916,7549) |
| Nanchang | 2010 | 632(164,1069) | 336(0,678) | 391(248,535) | 3806(2099,5434) | 1742(623,2796) | 6651(2314,10757) | 1355(502,2054) |
|  | 2015 | 1116(290,1878) | 575(0,1159) | 671(425,917) | 6579(3629,9387) | 3108(1112,4986) | 11233(3952,17971) | 2277(856,3413) |
|  | 2020 | 1299(337,2187) | 1130(0,2276) | 747(473,1020) | 7800(4303,11131) | 3355(1200,5382) | 12527(4400,20074) | 2541(953,3816) |
| Chongqing | 2010 | 6376(1659,10719) | 5412(0,10911) | 2923(1851,3992) | 35245(19447,50286) | 20299(7266,32556) | 119278(41856,191310) | 29171(10931,43848) |
|  | 2015 | 11792(3099,19646) | 10297(0,20661) | 5571(3531,7601) | 67536(37300,96265) | 36873(12427,55508) | 220616(79097,346655) | 53478(20575,78644) |
|  | 2020 | 13709(3607,22818) | 19652(0,39355) | 7024(4453,9579) | 74196(40507,104520) | 34197(12262,54752) | 274774(99380,428195) | 66367(25809,96747) |
| Chengdu | 2010 | 2389(619,4029) | 2416(0,4872) | 1304(826,1782) | 13473(7432,19228) | 6507(2328,10442) | 53390(18694,85813) | 13069(4884,19689) |
|  | 2015 | 4506(1178,7544) | 5492(0,11009) | 2973(1885,4055) | 24411(13475,34812) | 11971(4288,19184) | 116956(42127,182968) | 28296(10949,41419) |
|  | 2020 | 7775(2039,12929) | 11114(0,22294) | 3969(2516,5416) | 39081(21580,55718) | 23763(8516,38066) | 156942(56338,246313) | 38024(14651,55847) |

Supplementary Table A5 Health economic losses of PM_2.5_ pollution from vehicles in some cities (mean and 95% CI)

| Cities | Health economic loss/100 million yuan | | | the proportion of health economic loss to GDP/% | | | health economic loss per capita/yuan | | |
| --- | --- | --- | --- | --- | --- | --- | --- | --- | --- |
|  | 2010 | 2015 | 2020 | 2010 | 2015 | 2020 | 2010 | 2015 | 2020 |
| Beijing | 121.09(37.80,194.01) | 379.03(119.62,600.16) | 805.83(253.67,1263.14) | 0.86(0.27,1.37) | 1.53(0.48,2.42) | 2.24(0.70,3.51) | 617.16(192.66,988.84) | 1745.87(550.97,2764.45) | 3680.75(1158.70,5769.61) |
| Tianjin | 45.78(14.15,73.91) | 145.77(45.44,233.21) | 127.36(39.97,203.52) | 0.49(0.15,0.80) | 1.33(0.41,2.14) | 0.88(0.28,1.40) | 352.44(108.95,568.99) | 942.25(293.72,1507.48) | 918.36(288.19,1467.57) |
| Shijiazhuang | 22.42(6.95,36.14) | 68.72(21.56,109.42) | 57.20(17.61,92.00) | 0.65(0.20,1.06) | 1.61(0.51,2.57) | 0.96(0.29,1.55) | 220.34(68.29,355.15) | 642.18(201.48,1022.44) | 508.82(156.62,818.39) |
| Baoding | 15.98(4.97,25.74) | 58.68(18.59,92.86) | 55.83(17.69,88.72) | 0.78(0.24,1.26) | 1.78(0.56,2.81) | 1.41(0.45,2.24) | 142.57(44.35,229.64) | 507.94(160.89,803.79) | 483.57(153.25,768.41) |
| Cangzhou | 10.25(3.16,16.58) | 39.79(12.46,63.48) | 34.28(10.75,54.86) | 0.47(0.14,0.75) | 1.49(0.47,2.39) | 0.93(0.29,1.48) | 143.47(44.29,232.12) | 534.60(167.44,852.86) | 469.48(147.27,751.27) |
| Handan | 14.31(4.43,23.11) | 47.41(14.94,75.29) | 38.69(12.26,61.57) | 0.61(0.19,0.97) | 1.79(0.57,2.85) | 1.06(0.33,1.69) | 155.78(48.23,251.47) | 502.59(158.36,798.21) | 410.92(130.25,653.96) |
| Tangshan | 23.00(7.11,37.13) | 75.66(23.77,120.23) | 86.56(27.09,137.79) | 0.51(0.16,0.83) | 1.47(0.46,2.35) | 1.21(0.38,1.92) | 303.39(93.74,489.74) | 969.79(304.66,1541.13) | 1121.56(351.06,1785.36) |
| Wuhan | 46.24(14.22,74.53) | 114.70(35.51,183.30) | 154.79(47.57,248.21) | 0.83(0.26,1.34) | 1.05(0.33,1.68) | 0.99(0.30,1.59) | 417.31(128.28,672.62) | 1081.30(334.74,1727.97) | 1243.58(382.13,1994.03) |
| Changsha | 27.05(8.28,43.72) | 76.86(23.70,123.20) | 106.26(32.5,170.82) | 0.59(0.18,0.96) | 0.90(0.28,1.45) | 0.88(0.27,1.41) | 384.24(117.69,620.91) | 1034.26(318.94,1657.78) | 1056.20(323.55,1697.84) |
| Nanchang | 11.72(3.59,18.98) | 31.41(9.65,50.56) | 42.32(12.91,68.29) | 0.53(0.16,0.86) | 0.79(0.24,1.26) | 0.74(0.22,1.41) | 232.03(71.01,375.66) | 592.28(181.89,953.48) | 676.51(206.39,1091.83) |
| Chongqing | 120.84(39.77,190.49) | 368.16(123.53,571.05) | 615.10(208.85,947.37) | 1.49(0.49,2.34) | 2.27(0.76,3.52) | 2.41(0.82,3.71) | 418.92(137.88,660.35) | 1220.47(409.50,1893.06) | 1916.86(650.85,2952.31) |
| Chengdu | 78.53(26.12,123.49) | 227.61(78.63,349.27) | 376.10(126.84,582.34) | 1.41(0.47,2.22) | 2.11(0.73,3.23) | 2.12(0.72,3.29) | 559.01(185.93,879.11) | 1552.82(536.43,2382.85) | 1795.47(605.51,2780.06) |
